# BMP signalling differentially regulates distinct haematopoietic stem cell types

**DOI:** 10.1038/ncomms9040

**Published:** 2015-08-18

**Authors:** Mihaela Crisan, Parham Solaimani Kartalaei, Chris Vink, Tomoko Yamada-Inagawa, Karine Bollerot, Wilfred van IJcken, Reinier van der Linden, Susana M. Chuva de Sousa Lopes, Rui Monteiro, Christine Mummery, Elaine Dzierzak

**Affiliations:** 1 Department of Cell Biology, Erasmus MC Stem Cell Institute, Erasmus Medical Center, Wytemaweg 80, 3015 CN Rotterdam, The Netherlands; 2 University of Edinburgh, Centre for Inflammation Research, Queens Medical Research Institute, 47 Little France Crescent, Edinburgh EH16 4TJ, UK; 3 Center for Biomics, Erasmus Medical Center, Wytemaweg 80, 3015 CN Rotterdam, Netherlands; 4 Department of Anatomy and Embryology, Leiden University Medical Center, Building 2, Einthovenweg 20, 2333 ZC Leiden, The Netherlands

## Abstract

Adult haematopoiesis is the outcome of distinct haematopoietic stem cell (HSC) subtypes with self-renewable repopulating ability, but with different haematopoietic cell lineage outputs. The molecular basis for this heterogeneity is largely unknown. BMP signalling regulates HSCs as they are first generated in the aorta-gonad-mesonephros region, but at later developmental stages, its role in HSCs is controversial. Here we show that HSCs in murine fetal liver and the bone marrow are of two types that can be prospectively isolated—BMP activated and non-BMP activated. Clonal transplantation demonstrates that they have distinct haematopoietic lineage outputs. Moreover, the two HSC types differ in intrinsic genetic programs, thus supporting a role for the BMP signalling axis in the regulation of HSC heterogeneity and lineage output. Our findings provide insight into the molecular control mechanisms that define HSC types and have important implications for reprogramming cells to HSC fate and treatments targeting distinct HSC types.

## Abstract (web summary)

How bone morphogenetic proteins (BMPs) regulate haematopoietic stem cells (HSCs) later in development is unclear. Crisan *et al.* show that long-term repopulating HSCs in murine fetal liver and the bone marrow are of two types: either BMP activated or non-BMP activated, which correlate with different lineage outputs.

## Graphical abstract

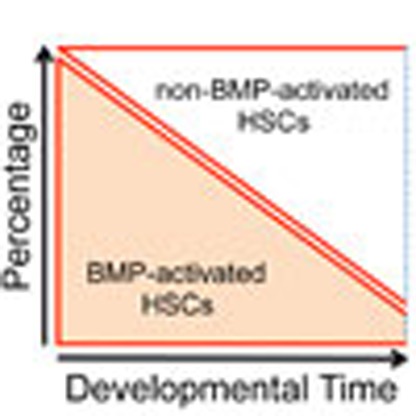

Understanding the signalling pathways and mechanisms by which haematopoietic stem cells (HSCs) sustain their robust homoeostatic and regenerative characteristics is important for disease treatments that may differentially affect HSC subtypes. Long-term repopulating HSC subtypes have been defined by their haematopoietic lineage output—myeloid–lymphoid balanced (Bala) HSCs, myeloid-biased (My) HSCs and lymphoid-biased (Ly) HSCs. The clonal composition of the HSC compartment is age-dependent^1^,^2^,^3^,^4^. Bala-HSCs are found throughout ontogeny, Ly-HSCs predominate in young individuals and My-HSCs predominate in older individuals^2^,^3^,^4^. The molecular basis for HSC subtypes is hindered by the lack of prospective isolation. Since bone morphogenetic protein (BMP) affects the development of HSCs in the embryo, HSC subtypes may be differentially controlled by the BMP signalling pathway. It is known that high concentrations of BMP4 maintain proliferation and repopulating activity of human cord blood HSCs^5^. However, it has also been shown that conditional inactivation of *BMPR1a* increases the number of bone marrow (BM) HSCs^6^, that the canonical BMP pathway is dispensable in embryonic day 14 (E14) fetal liver (FL) and adult BM^7^ and that inhibition of Smad-dependent BMP signalling enhances homing and engraftment of BM HSCs^8^. Since the role of BMP in HSC regulation is as yet uncertain, we examined whether HSCs in the embryo, fetus and adult are directly responsive to BMP signalling and the relationship of BMP signalling to HSC heterogeneity.

Here we show in BMP responsive element (*BRE) GFP* transgenic mouse embryos^9^ that all HSCs emerging *in vivo* in the aorta-gonad-mesonephros (AGM) region are BMP activated. In contrast, HSCs in murine FL and BM are of two types—BMP activated and non-BMP activated. The initially high proportion of BMP-activated HSCs decreases through ontogeny, and is surpassed by HSCs that are non-responsive to BMP. Clonal transplantation of the two BM HSC types demonstrates that HSC lineage output correlates with the state of BMP activation. Moreover, the two HSC types differ in intrinsic genetic programs thus supporting a role for the BMP signalling axis in the regulation of HSC heterogeneity and lineage output.

## Results

### All AGM HSCs *in vivo* are BMP-activated

**Figure 1:**
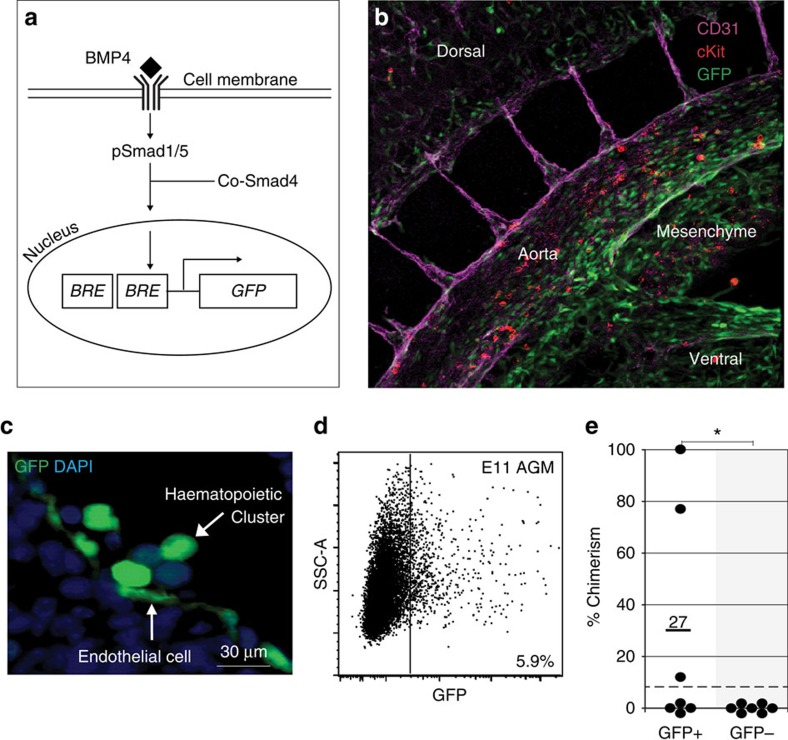
Aorta-gonad-mesonephros HSCs are BMP activated. (**a**) Scheme showing activation of the canonical BMP signalling pathway through phospo-Smad1/5 binding of the double BMP responsive element (*BRE*) and the resulting transcription of *GFP*. (**b**) Three-dimensional whole-mount image of an E10.5 immunostained *BRE–GFP* mouse embryo (37 somite pairs). Anti-CD31 (magenta), cKit (red) and GFP (green) antibody staining shows the predominantly ventral distribution of GFP^+^ cells in various cell types within the aorta and underlying mesenchymal cells. (**c**) High-magnification image of a transverse section of an E11 *BRE–GFP* dorsal aorta stained with anti-GFP-antibody and 4,6-diamidino-2-phenylindole (DAPI). Intra-aortic hematopoietic clusters contain GFP^+^ and GFP^−^ cells. Some endothelial cells are GFP^+^. (**d**) Representative FACS plot with side scatter on the *y* axis showing percentage of E11 AGM GFP^+^ cells (see Supplementary Fig. 1A for control). (**e**) Percentage donor cell chimerism in the peripheral blood of adult irradiated transplant recipients at 4 months after injection of E11 AGM GFP^+^ and GFP^−^ cells (2–4.5 embryo equivalents (ee) of AGM cells injected per mouse; *n*=3 independent transplantation experiments). *P=0.05 by z-Test for proportions.

The localized production of BMP4 in the AGM^10^,^11^,^12^ and *BMPR2* expression by enriched AGM haematopoietic progenitors and stem cells (HPSCs)^10^ suggests that BMP may act directly on HSCs. We examined the BMP activation status of HSCs during ontogeny in *BRE–GFP* transgenic mice. GFP is expressed in *BRE–GFP* mice when BMP and the BMP receptor signal through Smad1/5 to activate transcription from the *BRE* sequence (Fig. 1a). GFP expression denotes BMP activation at the moment of cell observation/isolation and does not represent previous BMP activation history. Importantly, all BRE*–*GFP-expressing cells also express pSmad1/5/8 (ref. 13). Three-dimensional imaging of whole-mount immunostained transgenic E10.5 embryos shows that BMP-activated (GFP^+^) cells are predominantly distributed in the ventral aspect of the aorta (lumenal hematopoietic, endothelial and underlying mesenchymal cells; Fig. 1b). HPSCs are known to reside in the haematopoietic clusters closely associated with the aorta^14^,^15^,^16^. Some cluster cells are GFP^+^ (Fig. 1c), suggesting heterogeneity in BMP activation within the HPSC compartment at the time of HSC generation. To test the BMP activation status of HSCs, E11 AGM GFP^+^ and GFP^−^ cells were sorted (6.2±3.1%(mean±s.d.) of E11 AGM cells are GFP^+^; Fig. 1d, Supplementary Fig. 1A) and transplanted into adult irradiated recipient mice. All AGM HSCs were found in the BMP-activated fraction (Fig. 1e): 3 out of 7 recipients receiving GFP^+^ AGM cells were long term, high level repopulated (>10% chimerism) by donor cells, whereas no recipients (0 out of 7) receiving GFP^−^ AGM cells were donor engrafted (*P*=0.05), even when high embryo equivalents of cells were injected. These data show that BMP signalling is activated in HSCs when they are first detected in the AGM.

### Two HSC types can be prospectively isolated from FL and BM

**Figure 2:**
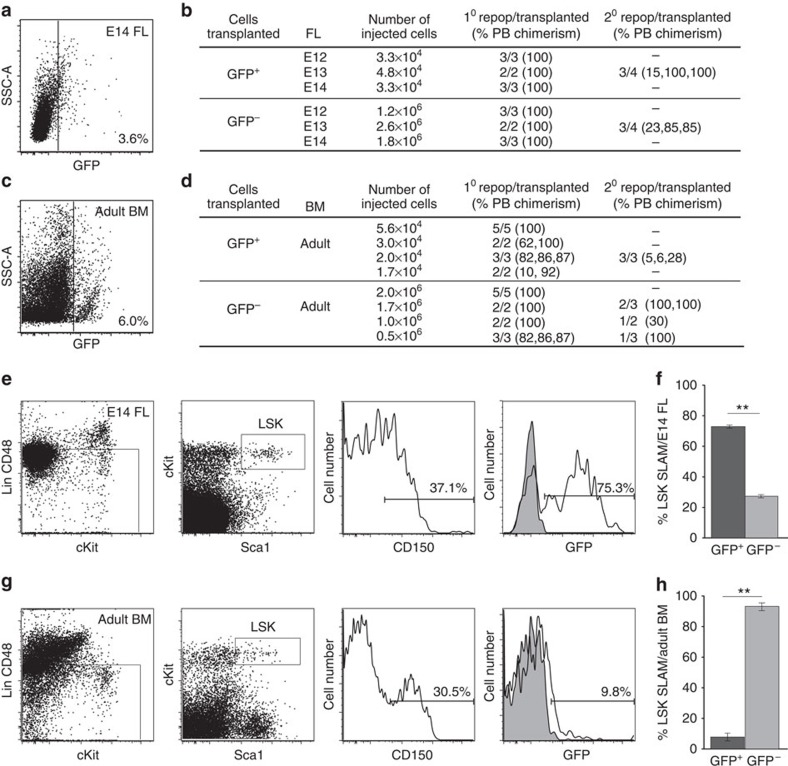
The fetal liver (FL) and bone marrow contain two distinct HSC types. Representative FACS plots showing percentage of GFP^+^ cells with side scatter shown on the *y* axis in the (**a**) E14 FL and (**c**) adult bone marrow (BM) of *BRE–GFP* mice (see Supplementary Figure S1A for controls). Transplantation results of adult irradiated recipients injected with GFP^+^ and GFP^−^ cells sorted from (**b**) E12, E13 or E14 FL (*n*=3) or (**d**) adult BM (*n*=4). Secondary irradiated adult recipients received BM cells, unsorted (3 × 10^6^) or GFP^+^ (1.5 × 10^5^) and GFP^−^ (0.8, 1.8 and 11.8 × 10^6^) sorted cells from repopulated primary recipients. Data from all secondary recipients were pooled. Peripheral blood (PB) donor chimerism is shown for all recipients. Representative FACS plots showing the sorting strategy for LSK-SLAM GFP^+^ and GFP^−^ cells from (**e**) E14 FL and (**g**) adult BM. Gates are shown for the Lin- and LSK fractions. Histogram analysis shows percentages of CD150^+^ cells in the LSK fractions and the percentages of GFP^+^ cells in the LSK-SLAM fractions of FL and BM. Grey curves show control non-transgenic LSK SLAM cells. Percentages of LSK SLAM cells that are GFP^+^ (black) or GFP^−^ (grey) as found in (**f**) E14 FL (*n*=3) and (**h**) adult BM (*n*=3). **P<0.001 *T*-test.

Examination of the BMP activation status of the cells in the *BRE–GFP* E14 FL and adult BM revealed that 3.7±0.5% (mean±s.d.) and 5.5%±1.8 (mean±s.d.) of cells, respectively, were GFP^+^ (Fig. 2a,c; Supplementary Fig. 1A). When sorted *BRE–GFP* E12*–*E14 FL or adult BM cells were transplanted, HSCs were found in both fractions, GFP^+^ and GFP^−^. Recipient mice were found to be long-term, high level repopulated (Fig. 2b,d) and secondary transplantation of BM from primary recipients showed that the HSCs self-renew (Fig. 2b,d). Thus, the FL and BM contain two types of HSCs—BMP activated and non-BMP activated. The percentage of phenotypic HSCs (LSK-SLAM; Lin^−^Sca1^+^cKit^+^CD150^+^CD48^−^) in E14 FL is significantly higher in the BMP-activated fraction (73%) than in the non-BMP-activated fraction (27%; *n*=5, *T*-test *P*<0.001; Fig. 2e,f). In contrast, in the BM, the majority (92%) of the phenotypic LSK-SLAM HSCs is non-BMP activated (Fig. 2g,h). BMP activation as detected by BRE*–*GFP expression allows prospective isolation of the two HSC types to enable study of their molecular and functional characteristics.

**Figure 3:**
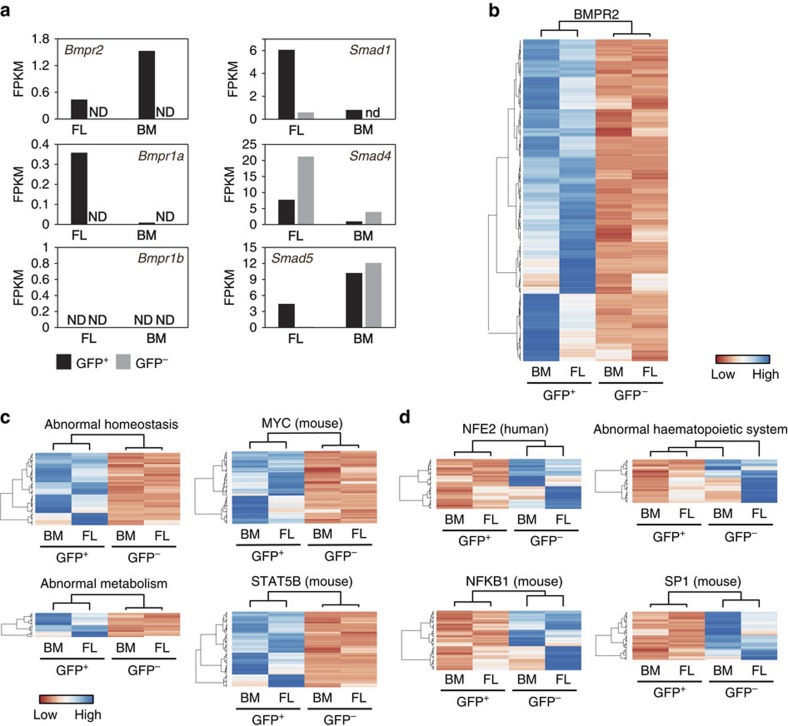
Transcriptome differences between BMP-activated and non-BMP-activated FL and BM HSCs. (**a**) FPKM values for *BMP* receptor and *Smad* genes expressed by GFP^+^ and GFP^−^ LSK SLAM sorted HSCs from E14 FL and adult BM. ND, not detected. (**b**–**d**) Gene list enrichment analysis on genes with more than two fold difference in expression level between GFP^+^ and GFP^−^ LSK SLAM sorted cells from FL and BM using *Enrichr* web-application (threshold false discovery rate (FDR)=0.01). GFP^+^ high genes are enriched in (**b**) BMPR2 gene targets (FDR<1.0e^−13^; Gene Expression Omnibus (GEO) Kinase perturbation gene sets). Gene lists available on request. (**c**) Also, overrepresented in GFP^+^ high genes are genes involved in homoeostasis (FDR=9.8e^−4^; Mouse Genome Informatics (MGI) mammalian phenotype term MP0001764), metabolism (FDR=5.4e^−4^; MGI mammalian phenotype term MP0005266), MYC (FDR=2.7e^−4^; TRANSFAC and JASPAR Position Weight Matrix (PWM) genesets), and STAT5B (FDR=2.7e^−4^; TRANSFAC and JASPAR PWM gene sets); (**d**) GFP^−^ high genes are enriched for NFE2 (FDR=9.6e^−3^; TRANSFAC and JASPAR PWM gene sets), NFKB1 (FDR=2.7e^−3^; TRANSFAC and JASPAR PWM gene sets), SP1 (FDR=2.7e^−3^; TRANSFAC and JASPAR PWM gene sets), and haematopoietic system (FDR=5.2e^−7^; MGI mammalian phenotype term MP0002396)-related genes.

The expression profiles of the BMP-activated and non-activated HSCs as examined by RNA sequencing show distinct genetic programs (Fig. 3). FL and BM LSK-SLAM GFP^+^ HSCs express *Bmpr* genes, whereas GFP^−^ LSK-SLAM HSCs do not (Fig. 3a). *Smad* genes are expressed in both fractions. Genes upregulated in the GFP^+^ FL and BM HSC fractions are significantly enriched in BMPR2 downstream targets, confirming BMP signalling activation (Fig. 3b). Moreover, other categories/gene sets were found to be differentially expressed between the two HSC types. For example, sets common to FL and BM upregulated in the BMP-activated HSCs are significantly enriched for genes involved in homoeostasis and metabolism, and MYC and STAT5B target genes (Fig. 3c). Upregulated sets in the non-BMP-activated HSCs are significantly enriched for genes involved in haematopoietic system development, NFKB1, SP1 and NFE2 target genes (Fig. 3d; see figure legend for false discovery rate corrected Fisher exact test *P* values). As some of these transcription factors affect normal and malignant haematopoietic cells and specific haematopoietic lineages, the distinct programs may influence the functional characteristics of the two HSC types.

### BM HSC output correlates with BMP activation status

**Figure 4:**
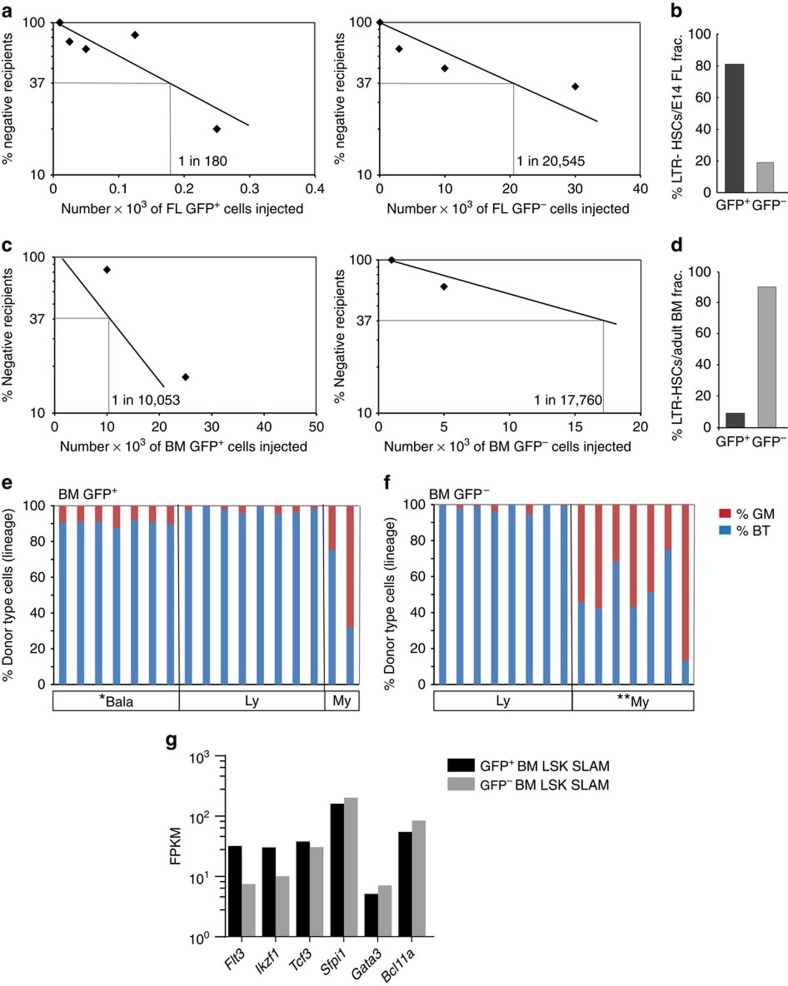
Relative percentages and lineage output of the BMP-activated and non-BMP-activated HSCs. Limiting dilution transplantation experiments show the frequencies of HSCs in GFP^+^ and GFP^−^ cell fractions from (**a**) E14 FL and (**c**) adult BM. See Supplementary Tables 1 and 2 for detailed results. Bar graphs showing percentages of long-term repopulating (LTR) HSCs that are GFP^+^ (black) or GFP^−^ (grey) in (**b**) E14 FL (*n*=5) and (**d**) adult BM (*n*=5) as calculated from limiting dilution transplantations. Summary of lineage output analysis of repopulated recipient mice injected with BM (**e**) GFP^+^ and (**f**) GFP^−^ cells at the clonal HSC level (*n*=3). Sorted GFP^+^ and GFP^−^ adult BM cells from 14–32-week-old *BRE–GFP* (Ly5.2) mice were injected intravenously into Ly5.1 irradiated (9 Gy) recipients and donor chimerism measured at 4 months post transplantation. Peripheral blood was analysed for donor Ly5.2 cell marker in granulocyte–macrophage (GM) lineages (Gr-1 and CD11b) and B- and T-cell lineages (B220 and CD3) by flow cytometry. Per cent donor contribution and donor percentage of GM and B+T were calculated according to Cho *et al.*^2^, to reveal HSC type in each recipient. *P* values were calculated by Fisher exact test between the GFP^+^ and the GFP^−^ fractions (*P*=0.007) and by two sample test for proportion (*χ*^2^) between balanced (*P=0.005), My (**P=0.031) and Ly-HSCs (*P*=0.727) of the two groups, GFP^+^ and GFP. (**g**) Fragments per kilobase of transcript per million mapped reads (FPKM) values for selected lymphoid genes expressed by GFP^+^ and GFP^−^ LSK SLAM sorted HSCs from adult BM.

Limiting dilution and clonal transplantations were performed to examine the frequency and functional properties of BMP-activated and non-activated HSCs. We found that a high frequency of FL GFP^+^ cells are HSCs (1 out of 180), whereas only 1 out of 20,545 FL GFP^−^ cells is a HSC (Fig. 4a; Supplementary Table 1). Given that the FL contains a mean of 12.7 × 10^6^ cells, of which 3.7% are GFP^+^, our data demonstrate that 81% of FL HSCs are BMP activated, with 19% being non-BMP activated (Fig. 4b). This corresponds well with the percentages of GFP^+^ and GFP^−^ cells found in the FL LSK-SLAM phenotypic HSC compartment. In contrast, the non-BMP-activated HSC number is significantly higher in the adult BM (Fig. 4c,d). One in 10,053 BM GFP^+^ cells and 1 in 17,760 BM GFP^−^ cells is an HSC (Supplementary Table 2). Taking into account an average of 2 × 10^7^ BM cells per mouse (four long bones), of which 5.5% are GFP^+^, we find that 9% of BM HSCs are BMP activated and 91% are non-BMP activated. This is in correspondence with the percentages of GFP^+^ and GFP^−^ cells found in the BM LSK-SLAM phenotypic HSC compartment. The inversed percentages of BMP-activated HSCs in the BM as compared with the FL are likely due to time- and/or niche-related ontogenic changes.

Considering this shift in the number of BMP-activated HSCs with developmental time and the data of others showing that there are changes in the clonal composition of the HSC compartment associated with lineage output^2^, we tested the lineage output of BMP-activated and non-activated HSCs. Clonal transplantations of GFP^+^ and GFP^−^ HSCs from FL and adult BM (injected cell number was calculated by limiting dilution experiments) were performed and the peripheral blood lineage output data at 4 months post-transplantation was analysed according to Cho *et al.*^2^ (Supplementary Fig. 1B). Only primary recipients that were reconstituted in both lymphoid and myeloid lineages were considered. Our data in the FL show slight differences in the lineage output composition of HSCs, with more Bala-HSCs in the BMP-activated fraction. However, this did not reach significance (Supplementary Fig. 1C,D; Supplementary Table 3). In contrast, BMP-activated and non-BMP-activated HSCs in the BM were significantly different in terms of HSC lineage output composition (*P*=0.007). Bala-HSCs were significantly enriched in the BMP-activated HSC fraction: 7 out of 17 in the GFP^+^ as compared with 0 out of 15 in the GFP^−^ fraction (*P*=0.005) (Fig. 4e; Supplementary Table 3). My-HSCs were significantly enriched in the non-BMP-activated fraction: 7 out of 15 in GFP^−^ compared with 2 out of 17 in the GFP^+^ (*P*=0.031; Fig. 4f; Supplementary Table 3). Ly-HSCs were equally distributed (8 out of 17 and 8 out of 15; *P*=0.727) (Fig. 4e,f; Supplementary Table 3). Thus, prospective isolation of predominantly balanced and myeloid-biased HSCs is achieved in the BM by cell sorting based on BMP activation.

### The two HSC types differ in their intrinsic molecular programs

Previously, it has been suggested that common lymphoid progenitor cells (CLPs) derived from clonal transplantation of lymphoid–myeloid HSCs are poised for expression of lymphoid regulator genes^17^. We examined whether the expression of such lymphoid genes are already present in LSK-SLAM HSCs and are associated with BMP activation status. Whereas 88% of GFP^+^ BM HSCs have enhanced lymphoid differentiation potential (Bala+Ly), this potential is present in only 53% of HSCs in the GFP^−^ fraction (Supplementary Table 3). Moreover, RNA sequencing data showed that the GFP^+^ BM HSCs have higher Fragments per kilobase of transcript per million mapped reads (FPKM) values for Ikaros (*Ikzf1*), E2A (*Tcf3*) and *Flt3* genes than GFP^−^ HSCs, supporting a pro-lymphoid transcriptional programme for BM BMP-activated HSCs. This is not observed in the FL where similar proportions of HSCs with enhanced lymphoid differentiation potential exist in both GFP^+^ (96%) and GFP^−^ (97%) fractions (Supplementary Table 3). FPKM values of the lymphoid genes are comparable in these fractions (Supplementary Fig. 1E). These data suggest that the BM microenvironment promotes a pro-lymphoid gene programme in subset of HSCs activated by the BMP signalling pathway. These results on HSC lineage output, together with the clear differences in the genetic programs between BMP-activated and non-BMP-activated HSCs, suggest the BMP signalling axis as an underlying basis for HSC heterogeneity.

## Discussion

The BMP activation marker that we used in this study represents a robust method by which functionally distinct types of HSCs can be prospectively enriched. Other methods previously used such as label retention^18^, ckit expression levels^19^, Hoechst dye efflux levels^20^ and CD229 provide limited separation^17^. It was shown that TGFβ1 differentially controls Ly- and My-HSCs^20^. However, the variation between the individual HSC clones found in this study suggests that HSC subtype segregation is not absolute. Indeed, as we show here only a subset of Ly-HSCs are controlled by the BMP pathway. Interestingly, TGFβ1 appears to be a general stimulatory factor for My-HSC proliferation^20^. This is in line with our study showing that in BM, in absolute number, there are significantly more My-HSCs in the non-BMP-activated fraction and these are likely to be controlled by the TGFβ pathway.

The ability to prospectively identify the distinct HSC types based on BMP activation and directly measure the clonal composition of the HSC pool highlights the importance of the BMP signalling axis in directing the intrinsic HSC programme during ontogeny. MYC and STAT5b target genes that are enriched in the BMP-activated HSCs are, respectively, known to be important in the balance between differentiation and self-renewal, and for conferring efficient lympho–myeloid repopulation and quiescence properties^21^,^22^. Of the (NFE2, SP1 and NFKB1) targets upregulated in the non-BMP-activated HSCs, NFE2 is often overexpressed in the myeloproliferative disorders, which spontaneously transform to acute myeloid leukaemia and polycythemia^23^. SP1 and NFKB1 are known to regulate HSC specification and are required for normal HSC function and differentiation^24^,^25^. Interestingly, aged HSCs express high levels of NFKB^3^,^26^. This is in line with our data showing that the non-BMP-activated BM HSCs are high in NFKB and are predominantly myeloid biased. These transcriptomic profiles together with further studies should provide insight into how HSC clonal composition may be regulated.

Our finding of the existence of non-BMP-activated HSCs now provides an explanation for the absence of a haematopoietic phenotype when the canonical BMP signalling pathway is impaired^6^,^7^,^8^. In the absence of BMP-activated HSCs, the non-activated HSCs are not only sufficient to assume the normal haematopoietic function, but may also expand. The earliest AGM HSCs are BMP activated and as development progresses and HSCs reside in other tissues, there is a shift in the ratio of HSC types from being predominantly BMP activated in the FL to being predominantly non-BMP activated in the adult BM. This implies quantitative changes in BMP-producing niches during the migration process from the AGM (BMP^high^) to FL and eventually multiple BM niches (osteoblastic, perivascular and neural) with variable BMP levels (BMP^high^ versus BMP^low^) or intrinsic changes in HSC BMP receptor expression with developmental time. Future studies should examine the specific niches supporting the two HSC types in the FL and BM for BMP production, and examine whether other signalling molecules regulate the non-BMP-activated HSCs. Deciphering the behaviour and lineage output of HSCs during ontogeny, understanding whether the two HSC types are interchangeable or have different origins and investigating the underlying intrinsic and extrinsic mechanisms that dictate these changes are of importance for ensuring that HSCs created through reprogramming strategies will be capable of generating all the desired cell types, and for understanding why specific haematological malignancies are prevalent in paediatric versus elderly patients^27^. Our findings may be of particular interest to drug resistance in acute myeloid leukaemia patients^28^, raising the question of whether a combination of drugs affecting the distinct HSC types would be more efficient to control blood-related disorders.

## Methods

### Mice and embryo generation

Mice were bred and housed at Erasmus MC. *BRE GFP* transgenic mice^9^ were maintained in C57BL/6 background. Two independently derived transgenic lines were used in this study. Data from line 1 are shown. Matings were set-up between heterozygous *BRE GFP* transgenic male and wild-type C57BL/6 female mice. The day of vaginal plug detection was designated as E0. All animal procedures were approved by the local ethics committee and performed in compliance with Standards for Care and Use of Laboratory Animals.

### Whole-mount immunostaining and immunohistochemistry

Whole-mount embryo immunostaining and three-dimensional imaging were performed as described previously^16^,^29^. Embryos were fixed for 20 min with 2% PFA/PBS at 4 °C; dehydrated in graded concentrations of methanol; stained with primary antibodies-unconjugated rabbit anti-GFP (MBL, 1:2000), biotinylated rat anti-CD31 (BD, 1:500) and subsequently with rat anti-cKit (BD, 1:500) antibodies in blocking buffer ON at 4 °C; washed; incubated with secondary antibodies goat anti-rabbit IgG-Alexa Fluor488 (Invitrogen, 1:1,000), goat anti-rat IgG-Alexa Flour647 (Invitrogen, 1:5,000) and donkey Streptavidin-Cy3, (Jackson ImmunoResearch, 1:1,000); made transparent in BABB; and analysed with a confocal microscope (Zeiss LSM 510META JNI, Plan-Neofluar × 10/0.3, Eppiplan-Neofluar × 20/0.50). Three-dimensional reconstructions were generated from z-stack with LSM Image Browser (Zeiss) and Amira (VISAGE IMAGING).

### Flow cytometry

AGMs were dissected with needles under the microscope as previously described^30^ and tissues were dissociated in PBS/FCS/PS with collagenase type I (Sigma, 0,125%) in water bath at 37 °C for 45 min. Cells were further mechanically separated by flushing with a pipette, washed with PBS/FCS/PS and centrifuged at 1,000 r.p.m. for 10 min at 4 °C. Pellets were resuspended in PBS/FCS/PS. E14 FL were mechanically disrupted until a homogeneous cell suspension was obtained. Adult BM was harvested from tibias and femurs by flushing with PBS/FCS/PS through a 1-ml syringe with a 25-G needle. Ficoll fractionation of BM cells was performed at 2,000 r.p.m., 20 min at room temperature. Mononuclear cells were harvested and washed with PBS/FCS/PS followed by centrifugation at 1,000 r.p.m., 10 min at 4 °C. Viable cells were counted by Trypan Blue exclusion (Sigma, 0.4%).

FL and adult BM LSK/SLAM cell stainings included LinPE cocktail (anti-CD3/1:400, anti-B220/1:2000, anti-Ter119/1:400, anti-Ly6c/1:4000 antibodies), anti-Sca1PE-Cy7(1:800), anti-cKitAPC-Alexa Fluor780(1:600) or BV421(1:500), anti-CD48PE(1:500) and anti-CD150APC(1:500) antibodies. Anti-CD11b-PE antibody (1:2,000) was added in the Lin staining for adult BM. Cells were analysed on a FACSAria SORP or FACSAria III (BD) with FloJo software. Dead cell exclusion was with Hoechst 33258 (1:10,000, Molecular Probes). Lineage output was tested on primary recipient peripheral blood at 4 months post transplantation using myeloid lineage (GM) markers, anti-CD11b PE and anti-Ly6c PE antibodies, lymphoid lineage (B+T) markers, anti-CD3APC(1:400) and anti-B220APC(1:2,000) antibodies and the donor marker with anti-CD45.2 FITC(1:500) antibody. All antibodies used were from BD Pharmingen except the anti-CD150 antibody (eBiolegend). Haematopoietic lineage output in peripheral blood of clonal transplant recipients (4 months post transplantation) was considered balanced when the B+T^value^/GM^value^/ was 4–16, myeloid-biased when the ratio was 0–4 and lymphoid-biased when the ratio was >16.

### Transplantation assay

Single cell suspensions were injected intravenously into female ((129SV × C57BL/6)F1 or Ly5.1) mice (3–6 months old) irradiated with 9 Gy (split dose, ^137^Cs source). A total of 2 × 10^5^ recipient background spleen cells were co-injected. BM cells from primary recipients were injected into irradiated (9 Gy, split dose) secondary recipients. For (129SV × C57BL/6) F1 recipients, peripheral blood peripheral blood chimerism was quantified by DNA PCR for *gfp* and calculated by DNA normalization (*myoD*) and comparison with *gfp* contribution controls with Image Quant software. Mice showing >10% donor chimerism were considered repopulated. For Ly5.1 recipients, peripheral blood chimerism was quantified by CD45.1 and CD45.2 flow cytometric analysis. Mice with >1% CD45.2^+^CD45.1^−^ donor chimerism were considered repopulated. Secondary recipients received either 3 × 10^6^ unsorted BM cells or GFP^+^ (1.5 × 10^5^ cells per mouse) and GFP^−^ (0.8−11.8 × 10^6^ cells per mouse) from the reconstituted primary recipients. For lineage output analysis, clonal HSC were transplanted based on frequencies found in the limiting dilution experiments. Briefly, GFP^+^ and GFP^−^ fractions from the BM and FL were first injected at different cell numbers in irradiated recipients and the repopulation activity measured at 4 months post injection. Using the following parameters, cell number transplanted and number of mice repopulated per number of mice transplanted, frequencies of HSCs were calculated with the L-Calc Software (StemCellTechnology). Subsequent transplants were performed with the cell numbers containing 1 HSC.

### RNA preparation and RNA sequencing

RNA was isolated from E14 and adult BM with the mirVana miRNA Kit and prepared according to SMARTER protocol^31^ for the Illumina HiSeq2000 sequencer. The sequencing depth (unique mapped reads) was for BM (GFP^+^ 1.21e^07^; GFP^−^ 1.11e^07^) and for the FL (GFP^+^ 1.22e^07^; GFP^−^ 9.63e^06^). Sequences were mapped to the mouse (NCBI37/mm9) genome and FPKMs were calculated using Bowtie (v2.2.3), TopHat (v2.0.12) and Cufflinks (v2.2.1). Differential expression was analysed using Cuffquant with fragment-bias and multi-read corrections and normalized across all samples using Cuffnorm with geometric library-size normalization^32^. Genes are ‘high in the BMP-activated HSC fraction (GFP^+^)' when FPKM in GFP^+^>2 × FPKM GFP^−^ in both BM and FL LSK-SLAM cells, and ‘high in the non-BMP-activated fraction (GFP^−^)' when FPKM in GFP^−^>2 × FPKM GFP^+^ in both BM and FL LSK-SLAM cells. For gene list enrichment analysis, genes were applied to *Enrichr* web application^33^. Gene sets with enrichment false discovery rate of 0.01 were selected and heatmaps (row scaled) were generated with ‘GFP^+^ high' or ‘GFP^−^ high' genes present in given enriched genelists.

## 

## Additional information

**Accession codes**: The RNA-seq data have been deposited in the Gene Expression Omnibus (NCBI) database with accession code GSE70132.

**How to cite this article:** Crisan, M. *et al.* BMP signalling differentially regulates distinct hematopoietic stem cell types. *Nat. Commun.* 6:8040 doi: 10.1038/ncomms9040 (2015).

## Supplementary Material

Supplementary Information Supplementary Figure 1, Supplementary Tables 1-3 and Supplementary Reference
