# Correction: Corrigendum: BMP signalling differentially regulates distinct haematopoietic stem cell types

**DOI:** 10.1038/ncomms9793

**Published:** 2015-10-29

**Authors:** Mihaela Crisan, Parham Solaimani Kartalaei, Chris S. Vink, Tomoko Yamada-Inagawa, Karine Bollerot, Wilfred van IJcken, Reinier van der Linden, Susana M. Chuva de Sousa Lopes, Rui Monteiro, Christine Mummery, Elaine Dzierzak

10.1038/ncomms9040

In the original version of this Article, the middle initial of the author Chris S. Vink was omitted from the author information. This has now been corrected in both the PDF and HTML versions of the Article.

