# Supplementary material for: BMP signalling differentially regulates distinct haematopoietic stem cell types
Source: Nat Commun. 2015 Aug 18;6:8040. doi: 10.1038/ncomms9040 (PMC4557333; doi:10.1038/ncomms9040)
Supplement: Supplementary Information — Supplementary Figure 1, Supplementary Tables 1-3 and Supplementary Reference [file ncomms9040-s1.pdf]

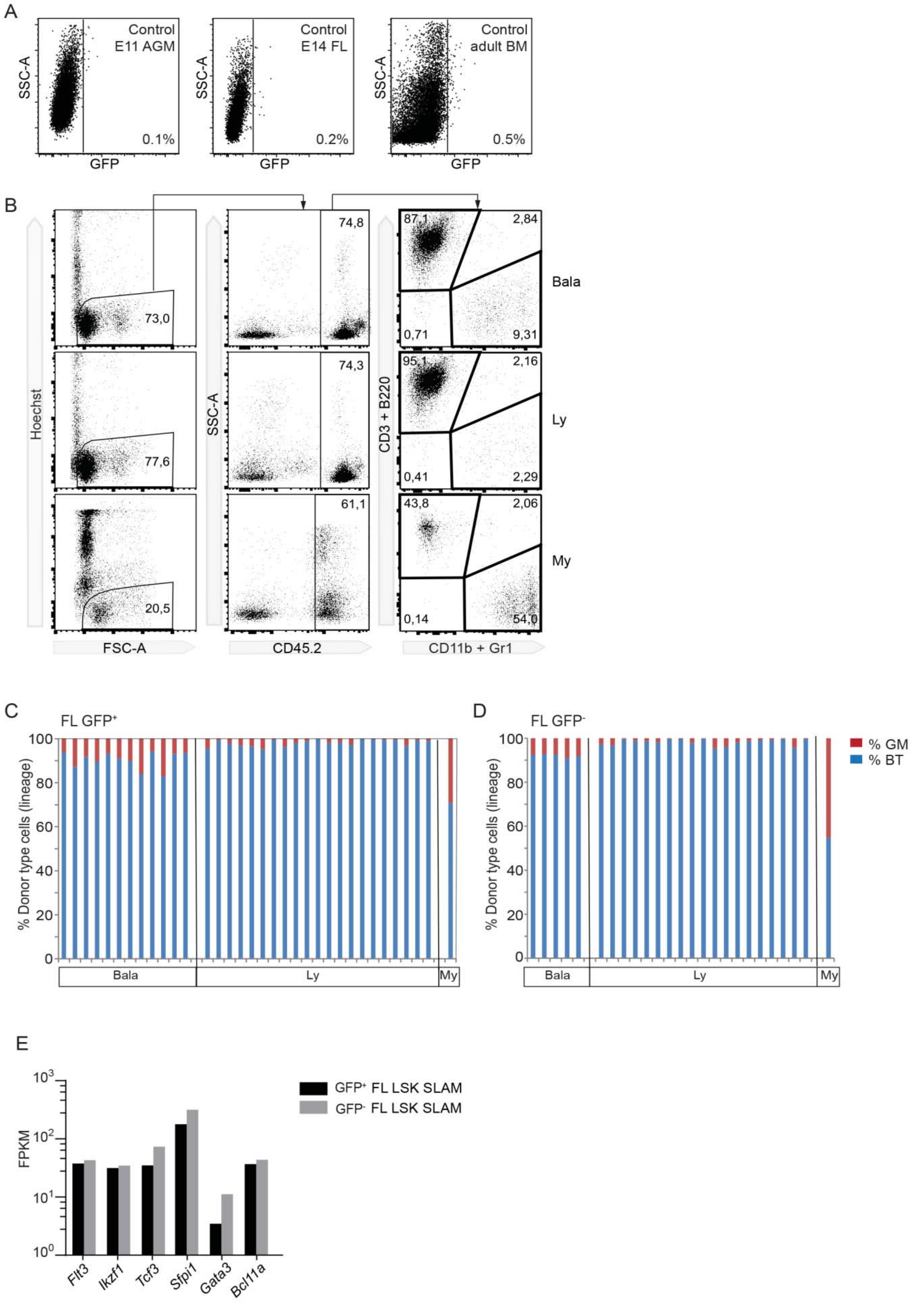

Figure S1

Supplementary Fig. 1

Supplementary Fig. **1**. **Analysis of lineage output composition and gene expression of the two hematopoietic stem cell types. (A)** Flow cytometric negative control from non-transgenic E11 AGM, E14 FL and adult BM. **(B)** Representative example of flow cytometric analysis of a myeloid-lymphoid balanced HSC (Bala, upper row), lymphoid-biased HSC (Ly, middle row) and myeloid-biased HSC (My, bottom row) . Sorted GFP^+ and GFP^- adult BM (from 14-32 week old) or E14 FL cells from *BRE-GFP* (Ly5.2) mice were injected intravenously into Ly5.1 irradiated (9Gy) recipients and donor chimerism measured at 4 months post-transplantation. Peripheral blood was analyzed for granulocyte-macrophage (GM) lineages (Gr-1 and CD11b) and B and T cell lineages (B220 and CD3) in the donor Ly5.2 cell marker expressing cells. Ratio between the Lymphoid (B+T) and Myeloid (GM) donor-derived GM and B+T (donor-derived) was calculated according to Cho et al[1] to reveal the clonal composition of the HSCs donor-derived in each recipient: My (0-4) Bala (4-16) and Ly (>16). Summary of lineage output analysis of repopulated recipient Ly5.1 mice (9Gy) injected with E14FL BRE-GFP Ly5.2 (C) GFP+ and (D) GFP- cells at the clonal HSC level (n=4). Donor chimerism and lineage output in peripheral blood was measured at 4 months post-transplantation. (E) FPKM values for selected lymphoid genes expressed by GFP^+ and GFP^- LSK SLAM sorted HSCs from E14 FL. SSC-A=side scatter analysis

SupplementaryTables

Supplementary **Table 1.** *In vivo* limiting dilution transplantation results of primary recipients receiving GFP^+ and GFP^- cells sorted from E14 fetal liver.

Cell Type	Cells ($x10^3$) injected	Repopulated/ transplanted mice	% Donor Chimerism	HSC Frequency	Average number HSCs/E14 FL
	3	5/5	100		
	1	3/3	43-100		
	0.5	3/3	85-100		
GFP^+	0.25	8/10	13-100	1/180*	2611**
	0.125	1/6	12		
	0.050	2/6	68-100		
	0.025	1/4	24		
	0.010	0/2	0		
	388	3/3	100		
	100	3/3	100		
	30	5/8	17-100		
	10	3/6	19-100		
GFP^-	6	2/6	22-100	1/20545*	595
	3	1/3	69		
	1	0/3	0		
	0.5	0/3	0		
	0.05	0/3	0		

* 95% Confidence Interval (lower/upper) frequency: for GFP^+HSC (1 in 309/1 in 104) and for GFP^- HSC (1 in 38314/1 in 11017), n=4.
** p = 0.0001
FL=fetal liver

Supplementary **Table 2.** *In vivo* limiting dilution transplantation results of primary recipients receiving GFP^+ and GFP^- cells sorted from adult bone marrow.

Cell Type	Cells ($x10^4$) injected	Repopulated/ transplanted mice	% Donor Chimerism	HSC Frequency	Average number HSCs/BM
	1	6/6	10-75		
	0.85	1/6	34		
GFP^+	0.65	2/6	11,40	1/10053*	110
	0.50	2/6	21,65		
	0.10	0/3	0		
	10	6/6	22-89		
	5	5/6	22-79		
GFP^-	2.50	5/6	5-27	1/17760*	1064
	1	1/6	11		

* 95% Confidence Interval (lower/upper) frequency: for GFP^+HSC (1 in 16995/1 in 5947) and for GFP^- HSC (1 in 32925/1 in 9580), n=3. BM=bone marrow

Supplementary **Table 3.** Lineage output HSC distribution (percentages and absolute number) in the E14 FL and BM (BRE-GFP^{+}, BRE-GFP^{-} and Total).

E14 FL	BRE GFP+			BRE GFP-			Total	
HSC subtype	subtype/total reconstituted	% subtype	Absolute cell no.	subtype/total reconstituted	% subtype	Absolute cell no.	Absolute cell no.	% subtype
Balanced	12/34	35	921	5/25	20	119	1042	33
Lymphoid	21/34	62	1612	19/25	76	452	2064	64
Myeloid	1/34	3	78	1/25	4	23	101	3
Total	34	100	2611	25	100	596	3207	100

BM	BRE GFP+			BRE GFP-			Total	
HSC subtype	subtype/total reconstituted	% subtype	Absolute cell no.	subtype/total reconstituted	% subtype	Absolute cell no.	Absolute cell no.	% subtype
Balanced	7/17	41	45	0/15	0	0	45	4
Lymphoid	8/17	47	52	8/15	53	567	619	53
Myeloid	2/17	12	13	7/15	47	497	510	43
Total	17	100	110	15	100	1064	1174	100

Supplementary Reference

1 Cho, R. H., Sieburg, H. B. & Muller-Sieburg, C. E. A new mechanism for the aging of hematopoietic stem cells: aging changes the clonal composition of the stem cell compartment but not individual stem cells. *Blood* **111**, 5553-5561, (2008).
